# Primary Thromboprophylaxis for the Prevention of Venous Thromboembolism in Cancer Patients with Central Venous Catheters: A Literature Review

**DOI:** 10.3390/jcm13061660

**Published:** 2024-03-14

**Authors:** Hikmat Abdel-Razeq, Mohammed J. Al-Jaghbeer

**Affiliations:** 1 Section of Hematology and Medical Oncology, Department of Internal Medicine, King Hussein Cancer Center, Amman 11941, Jordan; 2 School of Medicine, The University of Jordan, Amman 11941, Jordan; 3 Section of Pulmonary and Critical Care Medicine, Department of Internal Medicine, King Hussein Cancer Center, Amman 11941, Jordan; ma.16764@khcc.jo

**Keywords:** thromboprophylaxis, anticoagulants, venous thromboembolism, VTE, catheter related thrombosis, CRT, apixaban, rivaroxaban, LMWH

## Abstract

Cancer is a known risk factor for venous thromboembolism (VTE). The wider adoption of immunotherapy and anti-angiogenic drugs in recent years have increased this risk further. Central venous catheters (CVCs) are widely used access devices utilized to deliver infusion therapy, mostly in ambulatory settings. The endothelial injury associated with the use of these catheters adds to the risk of VTE to already high-risk patients. The introduction of direct oral anticoagulants (DOACs), with its proven efficacy and safety in multiple clinical indications, have renewed the attention to VTE prophylaxis in cancer patients with CVC. Several clinical trials and meta-analyses had shown that both apixaban and rivaroxaban are effective in lowering the risk of VTE, without increasing the risk of bleeding. Several risk assessment models (RAM) have utilized patient-related, tumor-related, and treatment-related factors, in addition to widely available biomarkers, like Hemoglobin (Hb) level, white blood cell (WBC) and platelets counts to stratify patients into two or three VTE risk levels. In this manuscript, we review the published clinical trials and meta-analyses that attempted to study the efficacy and safety of anticoagulants, mostly the DOACs, in cancer patients with CVCs. We will also propose a practical risk-directed approach to enhance VTE prophylaxis rate.

## 1. Introduction

The association between cancer and thrombosis is well known for over 200 years when Armand Trousseau (1801–1867) described what is now known as Trousseau’s syndrome or migratory thrombophlebitis, as a presenting feature of visceral cancer [1,2]. Almost one in five VTE events encountered in clinical practice is related to active cancer [3,4,5]. The incidence of VTE in cancer patients have significantly increased in recent years [6]. In one study, Danish medical registries were used to identify 499,092 patients with newly diagnosed cancer over a 20-year period between 1997 and 2017. Patients were matched to 1,497,276 non-cancer individuals from the general population. The risk of VTE in cancer patients was ninefold higher than in those without. Twelve-month incidence of VTE in the cancer cohort increased from 1.0% [95% Confidence Interval (CI), 0.9% to 1.2%] in 1997 to 3.4% (95% CI, 2.9% to 4.0%) in 2017 [7] and still increasing. Newer anti-cancer drugs, mostly anti-angiogenic agents and certain immunotherapy drugs, are associated with higher rates of thrombosis [8,9,10]. Such increase in the incidence of VTE in cancer patients can also be attributed to “better” survival rates observed in many cancer types, and “over utilization” of imaging studies which may uncover relatively high rate of asymptomatic “incidental” pulmonary embolism (PE) and deep vein thrombosis (DVT) [11,12]. Both components; PE and DVT are associated with increased morbidity and mortality in cancer patients and may delay highly needed anti-cancer therapy [13,14,15].

Central venous catheters (CVC), including peripherally inserted central catheters (PICC) lines and implanted CVC, are increasingly utilized in cancer patients, mostly to deliver infusional therapy for ambulatory patients. In addition to infection, CVC are known for their added risk of VTE [16,17,18]. Such increased risk may be related to endothelial cell injury and exposure of tissue factors, thus inducing a local (catheter-related thrombosis) or systemic clot; DVT and/or PE [19]. Catheter-related thrombosis (CRT) is a major clinical problem in terms of venous-access loss, risk of PE, and obviously the added cost [20,21,22,23]. This has resulted in multiple clinical societies guidelines recommending consideration of pharmacological primary thromboprophylaxis in ambulatory patients with cancer who are at high risk of VTE [24,25,26,27].

## 2. Methods

Published clinical trials, retrospective studies, and meta-analyses addressing the issue of thromboembolic complications and VTE prophylaxis following CVC insertion in patients with solid tumors were searched from PubMed using the following keywords: thromboprophylaxis, anticoagulants, venous thromboembolism, VTE, catheter related thrombosis, CRT, apixaban, rivaroxaban, low molecular weight heparin (LMWH), and warfarin.

Efficacy endpoints varied between studies and were reported as used in individual studies, such endpoints include: DVT, PE, VTE and CRT. When applicable, we also highlighted if the thrombus was symptomatic or asymptomatic. Safety endpoints included major bleeding, clinically relevant non-major bleeding (CRNMB) and minor bleeding. Thrombocytopenia was reported by some studies, especially those that utilized LMWH. Most studies defined major bleeding as bleeding that leads to death, symptomatic bleeding into vital organ, bleeding that mandates blood transfusion, or bleeding that leads to drop in hemoglobin level by >2 g/dL. CRNMB is defined as bleeding that is not major, but require any kind of medical intervention.

## 3. Results

### 3.1. Older Studies and Meta-Analyses

Many studies have tried vitamin-K antagonists (VKA) in VTE prophylaxis for cancer patients with CVC devices, however, all were relatively small and lacked the needed momentum, mostly related to higher rates of major bleeding and lack of survival advantage; an endpoint which was overemphasized in these studies [28]. Many of such studies were included in meta-analyses and will be discussed below.

Given the variations in number of patients enrolled, anticoagulant used, duration of treatment, and more importantly variation in end points followed by various studies, many researchers attempted to collect data from several published studies in meta-analyses. Cochrane team have published three updates; 2011 [29], 2014 [30] and 2018 [31] the last two will be discussed in more details below. More recently, researchers from University of Ottawa and McMaster University have published a more updated meta-analysis, too [32]. Three other older meta-analyses were reported by Carrier, et al. (2007) [33], Kirkpatrick, et al. (2007) [34] and Chaukiyal, et al. (2008) [35] will not be detailed here because studies included in these analyses were also included in the more recent meta-analyses addressed below.

#### 3.1.1. The Cochrane Meta-Analysis-2014

In this meta-analysis, published randomized clinical trials (RCT) and conference proceedings were searched from January 1966 to February 2013. Studies comparing the effects of thromboprophylaxis utilizing unfractionated heparin (UFH), LMWH, fondaparinux or VKA with placebo or observation were included. In total, 10 RCTs that enrolled 2564 adults with cancer and CVC, were included. Prophylactic-dose heparin, compared with no heparin, was associated with a statistically significant reduction in symptomatic DVT [Relative Risk (RR) 0.48; 95% CI 0.27 to 0.86]. However, results did not confirm or exclude a beneficial or detrimental effect of heparin on major bleeding (RR 0.49; 95% CI 0.03 to 7.84), or minor bleeding (RR 1.35; 95% CI: 0.62 to 2.92). Likewise, Low-dose VKA, compared with no VKA, was associated with a statistically significant reduction in asymptomatic DVT (RR 0.43; 95% CI 0.30 to 0.62). Compared with VKA, the use of heparin was associated with a statistically significant increase in asymptomatic DVT (RR 1.74; 95% CI 1.20 to 2.52) and thrombocytopenia (RR 3.73; 95% CI 2.26 to 6.16). However, results did not show or exclude a beneficial or detrimental effect on any of the other outcomes including symptomatic DVT and major bleeding. Details are described in Table 1 [30]. This meta-analysis included trials with all types of cancers at all stages, without taking into consideration any risk assessment model (RAM) or other risk stratification.

#### 3.1.2. The Cochrane Meta-Analysis-2018

This updated Cochrane meta-analysis included 13 RCTs with 3420 participants. Studies included examined the efficacy and safety of prophylactic-dose heparin (UFH or LMWH), or low-dose VKA (either fixed low dose or targeted INR of less than 2). However, none of the studies included in this meta-analysis used DOACs. Most studies administered the prophylactic anticoagulant for the specified fixed period or until CVC removal or thrombosis diagnosis. The studies varied in the thromboembolic outcomes; symptomatic and/or asymptomatic thrombosis; CVC-related or not related. In total six RCTs compared LMWH to no LMWH, five RCTs compared VKA to no VKA, and three others compared LMWH to VKA.

The analysis showed that the use of LMWH probably decreased the incidence of symptomatic catheter-related VTE compared to no LMWH (RR 0.43, 95% CI 0.22 to 0.81). However, authors stated that the analysis did not confirm or exclude a beneficial or detrimental effect of LMWH on mortality at three months of follow-up (RR 0.82, 95% CI 0.53 to 1.26), or major bleeding (RR 1.49, 95% CI 0.06 to 36.28). Analyses of the studies used VKA versus no VKA did not confirm or exclude a beneficial or detrimental effect of low-dose VKA compared to no VKA on mortality (RR 0.99, 95% CI 0.64 to 1.55), symptomatic catheter-related VTE (RR 0.61, 95% CI 0.23 to 1.64), or major bleeding (RR 7.14, 95% CI 0.88 to 57.78). Analysis of the studies that compared LMWH to VKA (Three RCTs, 641 participants) found no difference between LMWH and VKA on any of the end points, including mortality (RR 0.94, 95% CI 0.56 to 1.59), symptomatic VTE (RR 1.83, 95% CI 0.44 to 7.61), PE (RR 1.70, 95% CI 0.74 to 3.92) or major bleeding (RR 3.11, 95% CI 0.13 to 73.11). However, the meta-analysis showed that LMWH probably increased the risk of thrombocytopenia compared to VKA (RR 1.69, 95% CI 1.20 to 2.39), Table 1 [31].

#### 3.1.3. D’Ambrosio, et al. Meta-Analysis

In another, briefly reported meta-analysis, a total of 3018 patents, enrolled into 12 randomized clinical trials that compared thromboembolic prophylaxis (n = 1716) to placebo/observation (n = 1302). In this analysis, which focused on symptomatic VTE, anticoagulation, as compared with control, significantly reduced the risk of symptomatic VTE (RR 0.61, 95% CI, 0.42 to 0.88). The absolute incidence of VTE was reduced to 3.7% from 6.8%, *p* < 0.001. The number of patients needed to be treated (NNT) to prevent one event was 32 (95% CI, 21 to 65) [39].

#### 3.1.4. University of Ottawa and McMaster Meta-Analysis

**Figure 1:**
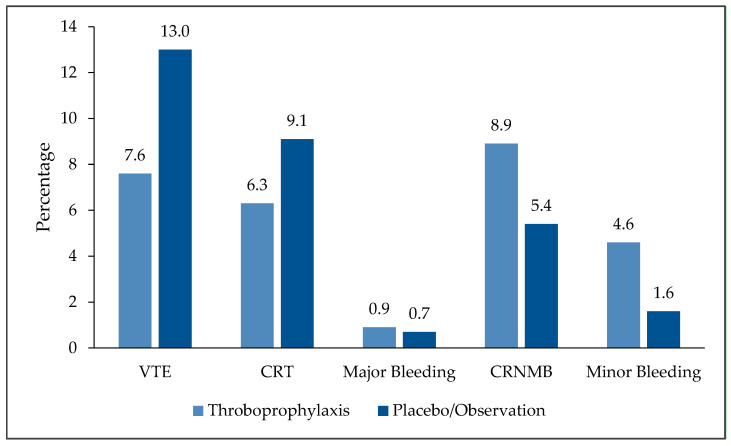
Efficacy and safety endpoints: Thromboprophylaxis versus placebo or observation. VTE: Venous thromboembolism; CRT: Catheter-related thrombosis; CRNMB: Clinically-relevant no major bleeding.

In this most recently published meta-analysis, RCTs that compared primary thromboprophylaxis using oral or parenteral anticoagulants, versus placebo or observation, among adult cancer patients with CVC were included. Both radiologically confirmed symptomatic and asymptomatic thromboembolic events, including CRT, were considered. In total, 3545 patients enrolled in 12 clinical trials were analyzed, this included three trials that were not part of the Cochrane meta-analyses. Five trials used VKA, five more used LMWH while three others used DOACs, and one trial used both LMWH and VKA. Both PICC lines and implanted CVC were used. Among the whole group, patients received VTE prophylaxis had lower incidence of VTE compared to those who did not; 7.6% versus 13.0% (OR 0.51, 95% CI 0.32 to 0.82, *p* < 0.01). Additionally, there were no significant differences in the rates of CRNMB bleeding (8.9% versus 5.4%; OR 1.28, 95% CI 0.81 to 2.04, *p* = 0.29) or major bleeding episodes (0.86% versus 0.65%; OR 1.12, 95% CI 0.29 to 4.40, *p* = 0.87). Minor bleeding, however, was reported more often in patients receiving thromboprophylaxis (4.6% versus 1.6%; OR 2.53, 95%CI 1.12 to 5.74, *p* = 0.03), Figure 1 [32].

### 3.2. Randomized Studies

#### 3.2.1. The AVERT Study

The AVERT trial was a randomized, placebo-controlled, double-blind clinical trial that was designed to test the safety and efficacy of apixaban in primary thromboprophylaxis for ambulatory cancer patients who were at intermediate to high risk of VTE as judged by the Khorana RAM (with a Khorana score of ≥2 indicating intermediate to high risk). Venous thromboembolism occurred in 4.2% of patients in the apixaban group and in 10.2% in the placebo group (HR, 0.41; 95% CI, 0.26 to 0.65; *p* < 0.001). Major bleeding, however, was reported in 3.5% in the apixaban group compared to 1.8% in the placebo group (HR, 2.00; 95% CI, 1.01 to 3.95; *p* = 0.046) [37].

In a subgroup analysis, reported separately, 217 ambulatory cancer patients had CVC and initiating chemotherapy; 126 were randomized to receive placebo, while 91 others received apixaban 2.5 mg orally twice daily. Confirmed VTE within 180 days of randomization, as a primary efficacy outcome, was reported in 18.7% in the placebo group, compared to only 4.80% among those who received apixaban, *p* < 0.0001. Major bleeding was not significantly different in the two groups; 1.6% versus 2.2%, *p* = 0.556 (Table 1) [38].

#### 3.2.2. TRIM-Line Pilot Trial

In another study, researchers at two Canadian centers conducted a prospective, randomized, blinded pilot trial that included 105 active cancer patients with newly inserted CVC. Patients were randomly assigned to receive thromboprophylaxis with rivaroxaban 10 mg daily or observation for 3 months. Overall, thrombotic complications were encountered in 3 (5.8%) patients in the rivaroxaban group compared and 5 (9.4%) patients in the control group (HR, 0.58; 95% CI, 0.14 to 2.5). Major VTE, defined as any symptomatic or incidentally detected proximal DVT of the lower or upper extremities, any fatal or nonfatal symptomatic or incidental PE, were encountered in 2 (3.9%; 95% CI, 0.5 to 13.2) and 3 (5.7%; 95% CI, 1.2 to 15.7) patients in the rivaroxaban and control group, respectively (HR, 0.66; 95% CI, 0.11 to 3.9). Among the whole group, only one patient (1.9%) in the rivaroxaban group, had a major bleeding event (Table 1) [39]. The TRIM-Line trial included a mix of cancer with different risks for VTE and did not apply a RAM for patient selection.

## 4. Who Is at Higher Risk?

Several investigators attempted to collect data on patient-related, [40] cancer-related and treatment-related factors to come up with risk assessment models that can stratify cancer patients initiating chemotherapy into different risk levels. The most widely recognized RAM is the one suggested by Khorana, et al. [41] the model was derived from a cohort of 2701 cancer patients and was validated internally on another independent cohort of 1365 patients. Five clinical predictive variables were included in this model: primary cancer, leukocyte count (WBC), platelet count, hemoglobin (Hb) level and/or use of erythropoiesis-stimulating agents (ESA), and body mass index (BMI), Table 2. According to the calculated scores, patients were stratified into 3 risk levels; low, intermediate and high-risk. The Khorana score may be limited in certain common types of cancer, like lung cancer [42,43].

In an effort to improve on VTE risk stratifications, our group proposed a new RAM (COMPASS-CAT) for ambulatory cancer patients with breast, colorectal, lung, and ovarian cancers. The COMPASS-CAT RAM includes variables related to the cancer itself (disease stage and time since cancer diagnosis), treatment-related variables (anthracycline or hormonal therapy), and patient-related factors and comorbidities (presence of cardiovascular risk factors, recent hospitalization for acute medical illness and personal history of VTE). Similar to Khorana RAM, the COMPASS-CAT also included biomarkers (platelet count). But contrary to Khorana’s RAM, the presence of CVC was added to the risk factors. Patients were then grouped into two (not three) risk categories; low/intermediate and high-risk groups, Table 3 [44].

For patients with lymphoma, Antic and colleagues introduced a new risk assessment model (ThroLy) specifically for patients with different types of lymphomas. The model includes history of prior venous or arterial thrombosis (including myocardial infarction and stroke), extranodal disease, mediastinal involvement, poor performance status (PS), obesity, low hemoglobin and low neutrophil counts. Based on calculated risk scores, patients were divided into three risk groups: low (score 0–1), intermediate (score 2–3), and high (score > 3) [45,46]. Our group utilized the International Prognostic Index (IPI), a simple tool that depends on clinicopathological variables including age, lactate dehydrogenase (LDH), number/sites of involvement, stage and patients’ PS to help predict both response to treatment and prognosis of patients with newly diagnosed diffuse large B-cell lymphoma (DLBL) [47,48]. Utilizing the IPI, we were able to stratify patients with DLBL into three risk levels for VTE [49].

In another retrospective study that included 177 patients who developed CVC complications among a cohort of 3046 ambulatory cancer patients, authors had clearly shown a strong association between catheter-related thrombosis and high-risk groups in Khorana (*p* = 0.0195), Protecht (*p* = 0.0412) and COMPASS-CAT (*p* = 0.0027) risk assessment models [50].

## 5. Discussion

Previous systematic reviews and meta-analyses have emphasized the importance of CVC as an independent risk factor for symptomatic VTE among patients with cancer on active chemotherapy. These meta-analyses, have also reported that the use of LMWH or VKA as primary thromboprophylactic agents were associated with a significant reduction in symptomatic VTE. Due to the inconvenience of daily injections with LMWH, difficulty in managing VKA in cancer patients, and the potential risk of bleeding complications among this high-risk patient population, these findings were never incorporated in routine clinical practice [51,52,53].

The introduction of DOACs with its convenient once or twice daily oral administration, and their previously reported efficacy and safety, when used to treat active VTE in cancer patients, can be viewed as an opportunity to enhance VTE prophylaxis in a subset of ambulatory cancer patients on active anti-cancer therapy utilizing a central venous catheter. Given the lack of “strong evidence” to offer thromboprophylaxis for each cancer patient with CVC, we are suggesting to use existing risk assessment models to select “higher-risk” ambulatory cancer patients, and offer them thromboprophylaxis, using DOACs, if they have CVC. Risk of bleeding and patients’ desire should also be considered. Above suggestions are in-line with the most recently updated recommendations endorsed by many professional international societies including the American Society of Hematology (ASH) [24], the American Society of Clinical Oncology (ASCO) [25], the European Society of Medical Oncology (ESMO) [26] and the International Society on Thrombosis and Hemostasis (ISTH) [27].

## 6. Future Directions

In an effort to maximize the benefit of thromboembolic prophylaxis against CRT, without increasing the risk of bleeding, researchers are trying new targets against coagulation factors FXI and FXII in many clinical indications including CRT [54,55]. In one exploratory study, 11 ambulatory cancer patients undergoing central line placement were given a single dose of gruticibart, an anti-FXI monoclonal antibody administered through the venous catheter within 24 h of placement. Patients were followed up by a surveillance ultrasound at day 14 for evaluation of catheter thrombosis. Another cohort of 11 patients on a parallel, noninterventional study was used as a comparator. The overall incidence of CRT was significantly lower (12.5%) in the interventional study compared to 40.0% in the parallel control study, *p* < 0.001. The drug was well tolerated and without clinically relevant bleeding or infusion reactions [54].

## 7. Conclusions

Almost half of VTE in cancer patients are encountered in ambulatory settings where VTE prophylaxis is not routinely practiced. With our efforts to move cancer care to ambulatory settings, CVC are increasing used to deliver infusional anti-cancer therapy; both are well known to further enhance the risk of VTE in cancer patients. Though VTE prophylaxis in patients with CVC is not routinely recommended, we believe a subset of ambulatory cancer patients identified to be at “higher risk”, based on available RAM, may be considered for thromboprophylaxis. Risk of bleeding and patient’s desire should also be strongly considered. Randomized clinical trials using DOACs in a risk-directed approach are highly needed.

## Figures and Tables

**Table 1:** Summary of published meta-analyses and clinical trials.

Study	Study Group(s)	Pulmonary Embolism	Symptomatic VTE	Major Bleeding	CRNMB	Minor Bleeding
Cochrane Meta-analysis 2014 [30]	LMWH versus No LMWH		0.48 *	0.49		1.35
			0.27–0.86 **	0.03–7.84		0.62–2.92
			(n = 1317) #	(n = 1012)		(n = 544)
	VKA versus no VKA		0.51	7.60		3.14
			0.21–1.22	0.94–61.49		0.14–71.51
			(n = 1451)	(n = 979)		(n = 979)
	LMWH versus VKA	1.70	2.15	3.41		0.95
		0.74–3.92	0.65–71.14	0.15–79.47		0.20–4.61
		(n = 317)	(n = 551)	(n = 279)		(n = 234)
Cochrane Meta-analysis 2018 [31]	LMWH versus No LMWH (n = 1537)		0.43	1.49		1.35
			0.22–0.81	0.06–36.28		0.62–2.92
			(n = 1089)	(n = 1018)		(n = 544)
	VKA versus no VKA (n = 1599)		0.61	7.14		0.69
			0.23–1.64	0.88–57.78		0.38–1.26
			(n = 1271)	(n = 1026)		(n = 1026)
	LMWH versus VKA (n = 641)	1.70	1.83	3.11		0.95
		0.74–3.92	0.44–7.61	0.13–73.11		0.20–4.61
		(n = 327)	(n = 327)	(n = 289)		(n = 234)
University of Ottawa and McMaster [32]	Thromboprophylaxis using oral or parenteral anticoagulants, versus placebo or observation (All patients, n = 3545)		0.51	1.12	1.28	2.53
			0.32–0.82	0.29–4.40	0.81–2.04	1.12–5.74
AVERT 2019 Apixaban study (Part of AVERT) [36,37]	Apixaban Versus Placebo Subgroup with CVC (n = 217)		0.26	0.69		
			0.14–0.47;	0.20–2.35;		
			*p* < 0.0001	*p* = 0.556		
TRIM-Line Pilot trial 2021 [38]	Rivaroxaban versus observation (n = 105)		0.58	Occurred in one patient (1.9%) compared to none	1.02	
			0.14–2.5		0.14–7.24	

VTE: Venous thromboembolism; CRNMB: Clinically relevant non-major bleeding; LMWH: Low-molecular weight heparin; VKA: Vitamin K antagonist; CVC: Central venous catheter. *: Risk Ratio (RR); **: 95% Confidence Interval (CI); #: Number of patients available for analysis for this end point.

**Table 2:** Khorana risk assessment model.

Patient Characteristic	Risk Score
Site of cancer	
Very high risk (stomach, pancreas)	2
High risk (lung, lymphoma, gynecologic, bladder, testicular)	1
Body Mass Index (BMI): ≥35 kg/m^2^	1
Biomarkers	
Prechemotherapy platelet count ≥350 × 10^9^/L or more	1
Hemoglobin < 100 g/L, or use of red cell growth factors	1
Prechemotherapy leukocyte count >11 × 10^9^/L	1

Risk groups: low risk (0); Intermediate risk (1–2); High risk (≥3).

**Table 3:** COMPASS-CAT risk assessment model.

Predictors for VTE	Score
Cancer-related risk factors	
Anti- hormonal therapy for women with HR-positive breast cancer, or anthracycline treatment	6
Time since cancer diagnosis ≤ 6 months	4
CVC	3
Advanced stage of cancer	2
Predisposing risk factors	
Cardiovascular risk factors, composed by at least two of the following predictors: - Personal history of peripheral artery disease, ischemic stroke, CAD - Hypertension, hyperlipidemia, diabetes, Obesity	5
Recent hospitalization for acute medical illness	5
Personal history of VTE	1
Biomarkers	
Platelets count ≥350 × 10^9^/L	2

HR: Hormone receptors; CVC central venous catheter; VTE venous thromboembolism; CAD: Coronary artery disease. Risk stratification: Low/intermediate risk (0–6); High risk (≥7).

## Data Availability

Not applicable.
